# AK098656: a new biomarker of coronary stenosis severity in hypertensive and coronary heart disease patients

**DOI:** 10.1186/s13098-022-00783-3

**Published:** 2022-01-15

**Authors:** Xin Wang, Ya-li Wu, Yuan-yuan Zhang, Jing Ke, Zong-wei Wang, Bao-yu Zhang, Yan Ma, Long-yan Yang, Dong Zhao

**Affiliations:** 1 Center for Endocrine Metabolism and Immune Diseases, Beijing Luhe Hospital, Capital Medical University, Beijing, China; 2 Beijing Key Laboratory of Diabetes Research and Care, Beijing, China

**Keywords:** AK098656, Coronary heart disease, Hypertension, Complications, Stenosis

## Abstract

**Background:**

AK098656 may be an adverse factor for coronary heart disease (CHD), especially in patients with hypertension. This study aimed to analyze the effect of AK098656 on CHD and CHD with various complications.

**Methods:**

A total of 117 CHD patients and 27 healthy control subjects were enrolled in the study. Plasma AK098656 expression was determined using the quantitative real-time polymerase chain reaction. Student’s *t*-test was used to compare AK098656 expression levels in different groups. Receiver operating characteristic (ROC) curve and area under the curve (AUC) were used to quantify the discrimination ability between CHD patients and health controls and between CHD and CHD + complications patients. The relationship between AK098656 and coronary stenosis was analyzed using Spearman’s correlation.

**Results:**

AK098656 expression was remarkably higher in CHD patients than in healthy controls (P = 0.03). The ROC curve revealed an effective predictive AK098656 expression value for CHD risk, with an AUC of 0.656 (95% CI 0.501–0.809). Moreover, AK098656 expression was increased in CHD + complications patients compared to CHD patients alone (P = 0.005), especially in patients with hypertension (CHD + hHTN, P = 0.030). The ROC curve revealed a predictive AK098656 prognostic value for discriminating between CHD and CHD + hHTN patients, with an AUC of 0.666 (95% CI 0.528–0.805). There was no significant difference in AK098656 expression in CHD patients with diabetes mellitus compared to CHD patients alone. In addition, AK098656 expression in CHD patients was positively correlated with stenosis severity (R = 0.261, P = 0.006).

**Conclusion:**

AK098656 expression was significantly increased in patients with CHD, especially those with hypertension, and its expression level was positively correlated with the degree of coronary stenosis. This implied that AK098656 may be a risk factor for CHD and can potentially be applied in clinical diagnosis or provide a novel target for treatment.

## Introduction

Cardiovascular disease is the leading cause of death worldwide. Coronary heart disease (CHD) is the most harmful disease and leading cause of hospitalization and death among adult heart disease patients [1–3]. Multiple studies have shown that controlling the risk factors can significantly reduce CHD morbidity and mortality [4–8]. Many traditional risk factors, such as family history of CHD, hypertension, diabetes, obesity, and smoking, already have been targeted for early interventions in clinical work [9, 10]. However, current research has shown that some nontraditional factors, such as autoimmune diseases, high sensitivity C-reactive protein, and homocysteine, also play important roles in the occurrence and development of CHD and need to be given sufficient attention.

Long non-coding (lnc) RNA has a length of more than 200 nucleotides and limited protein-coding ability and can be considered as “transcriptional junk” [11]. However, growing evidence has suggested that lncRNA has important regulatory functions and is associated with the development of complex diseases, such as cardiovascular diseases and cancer [12–15]. Accumulating studies have revealed that multiple lncRNAs are implicated in CHD. An earlier study has shown that lncRNA-p21 enhances p53 activity via the p53 signaling pathway, thereby inhibiting the formation of atherosclerosis [16]. CoroMarker is very stable in the plasma of CHD patients and may become a new plasma biomarker for CHD diagnosis [17]. H19 is re-expressed in atherosclerotic patients, and its polymorphism is related to the risk of CHD [18].

A new human vascular smooth muscle cells (VSMCs)-dominant lncRNA AK098656 can accelerate the degradation of contractile proteins, increase the synthesis phenotype in VSMCs, and eventually lead to narrowing of resistant arteries and hypertension [19]. VSMCs phenotype conversion has crucial pathophysiological significance in different stages of atherosclerosis, while lncRNAs are important factors that regulate VSMCs function and phenotype, as well as occurrence and development of related diseases. However, because AK098656 is a newly discovered lncRNA specifically expressed in VSMCs, its relationship with CHD has not been reported. In this study, we will sought to elucidate that AK098656 is a new molecule that regulates the development of coronary heart disease.

## Materials and methods

### Patients and samples

Due to the limited data on AK098656 expression in CHD patients, plasma and clinical data for healthy controls (n = 27) and CHD patients (CHD: n = 25, CHD + hDM: n = 24, CHD + hHTN: n = 41, and CHD + hDM + hHTN: n = 27) were collected in Beijing Luhe Hospital, Capital Medical University (Beijing, China) between September 2019 and January 2020. All participants were Han individuals from the same region. Patient inclusion criteria were as follows: (1) patients diagnosed with CHD. CHD was defined by coronary artery stenosis of ≥ 50% as determined by coronary angiography. Hypertension was defined by systolic blood pressure of at least 140 mm Hg or diastolic blood pressure of at least 90 mm Hg. Diabetes was defined by fasting blood glucose level of at least 7.0 mmol/L or blood glucose level of 11.0 mmol/L or more 2 h after an oral glucose load of 75 g; and (2) age of 35–80 years. Exclusion criteria included other clinically detectable cardiovascular diseases. The study complied with the Helsinki Declaration for investigation of human subjects and obtained ethical approval from the competent Institutional Review Boards of Beijing Luhe Hospital, Capital Medical University. All participants provided written informed consent.

### RNA isolation and qPCR

Blood samples of patients were collected within 24 h after admission to the Cardiology Intensive Care Unit. After centrifugation at 5000*g* for 10 min, the plasma was separated from the whole blood and divided into two portions. All samples were stored at − 80 °C. Total RNA from human plasma was isolated using the TRIzol LS (Invitrogen, USA) and converted to cDNA using a cDNA synthesis kit (BioRad, USA) according to manufacturer instructions. The expression levels of lncRNA-AK098656 were determined using SYBR (BioRad, USA) and normalized using 18s cDNA. The primer sequences were as follows: AK098656, forward: 5'-CCTCATTTGCTGGCACCTG-3', reverse: 5'-GGGAGGCAAGGTAGAAGGGA-3'; 18S, forward, 5'-GTAACCCGTTGAACCCCATT-3', and reverse: 5'-CCATCCAATCGGTAGTAGCG-3'.

### Biochemical measurements

Blood samples were collected for triglyceride, cholesterol, and high- and low-density lipoprotein cholesterol analysis using a Hitachi 7600-020 clinical analyzer (Hitachi, Tokyo, Japan). C-peptide and insulin are determined by Cobas e601 (Roche Diagnostics, Mannheim, Germany).

### Coronary angiography

Coronary angiography methods have been previously described [20]. Patients with coronary artery stenosis ≥ 50% were diagnosed with CHD. CHD severity was further divided into three groups: mild, moderate, and severe. Mild refers to the degree of stenosis < 70% for all coronary arteries, moderate refers to the degree of stenosis ≥ 70% for one coronary artery, and severe refers to the degree of stenosis ≥ 70% for two or more coronary arteries.

### Statistical analysis

To explore the relationship between AK098656 and CHD, Student’s *t*-test between healthy controls and CHD groups was used. Furthermore, receiver operating characteristic (ROC) curve was utilized to estimate the discrimination ability of AK098656 in CHD and to quantify the discrimination ability using an area under the curve (AUC), which is widely used in pattern recognition. The distribution of AUC values was between 0 and 1. AUC > 0.5 meant that the indicator had the ability to distinguish between conditions in question. The larger the AUC value, the stronger the ability to distinguish.

Student’s *t*-test was then used between the CHD and CHD + complications groups (CHD + hDM, CHD + hHTN, and CHD + hDM + hHTN, n = 92) to explore the relationship between AK098656 and complications with CHD. The ROC curve and AUC were used to quantify the discrimination ability between CHD and CHD + complications. In addition, Student’s *t*-test was utilized between the CHD and CHD + hDM groups and with the CHD + hHTN group separately to explore which complication would result in a greater effect on AK098656. The ROC curve and AUC were also used to quantify the differences.

Hypertension is one of the most potent risk factors for CHD development, which increases the risk of stenosis in CHD patients. The correlation between AK098656 and CHD stenosis was evaluated using Spearman’s correlation analysis.

## Results

### General characterization of study subjects

Table 1 provides the general characteristics for participants with or without CHD and CHD accompanied by complications. The study included 27 non-CHD participants with an average age of 49 years (13 men). For CHD patients (19 men), the average age was 64 years. Compared to healthy controls, CHD and CHD + complications patients had a higher heart rate and increased systolic blood pressure. CHD patients diagnosed with diabetes had a higher fasting glucose level.

**Table 1:** Demographics and clinical characteristics

Characters	Healthy controls (n = 27)	CHD (n = 25)	CHD + hDM (n = 24)	CHD + hHTN (n = 41)	CHD + hDM + hHTN (n = 27)
Age, years	48.9 ± 14.9	62.2 ± 13.8	63.0 ± 10.4	62.4 ± 11.4	68.3 ± 11.3
Gender, M/F	13/14	19/6	13/11	29/12	15/12
Heart rate, beats/min	60.7 ± 41.9	73.8 ± 15.6	81.9 ± 17.2	73.8 ± 17.4	81.9 ± 21.0
Hypertension history	No	No	No	Yes	Yes
Drug use					
Aspirin, n (%)	3 (11.1%)	22 (88%)	24 (100%)	33 (80.5%)	21 (77.8%)
Clopidogrel, (%)	0	9 (36%)	15 (62.5%)	18 (43.9%)	15 (55.6%)
Statin, n (%)	0	17 (68%)	23 (95.8%)	35 (85.4%)	20 (74.1%)
Calcium antagonist, (%)	4 (14.8%)	2 (8%)	2 (8.3%)	17 (41.5%)	13 (48.1%)
β-blocker, n (%)	3 (11.1%)	13 (52%)	16 (66.7%)	24 (58.5)	12 (44.4%)
Total cholesterol, mmol/L (SCHO)	4.48 ± 0.88	4.78 ± 1.21	4.64 ± 1.09	4.21 ± 1.12	3.93 ± 0.89
Total trigly (STG)	1.19 ± 0.71	1.62 ± 0.87	1.94 ± 1.28	1.50 ± 0.80	1.65 ± 0.97
HDL cholesterol, mmol/L (SHDL)	1.37 ± 0.39	1.14 ± 0.25	1.06 ± 0.24	1.09 ± 0.23	1.12 ± 0.24
LDL cholesterol, mmol/L (SLDL)	2.67 ± 0.67	3.09 ± 0.84	3.15 ± 0.83	2.73 ± 0.90	2.42 ± 0.74
Fasting glucose, mmol/L (SFBG)	5.49 ± 1.20	5.55 ± 0.99	8.54 ± 3.37	5.77 ± 1.37	8.98 ± 2.99
Insulin	10.96 ± 6.69	14.75 ± 14.41	18.09 ± 20.80	17.83 ± 16.40	24.00 ± 27.50
C-peptide	2.35 ± 0.69	3.76 ± 12.71	3.78 ± 4.01	3.87 ± 2.08	4.25 ± 3.07
Coronary stenosis					
Mild	–	4	2	4	3
Moderate	–	7	4	14	5
Severe	–	8	8	12	8
Not available	–	6	10	11	11

Data are presented as mean value ± standard deviation or count (%). CHD: coronary heart disease; hDM: human diabetes mellitus; hHTN: human hypertension

### AK098656 expression correlation for CHD risk

**Fig. 1:**
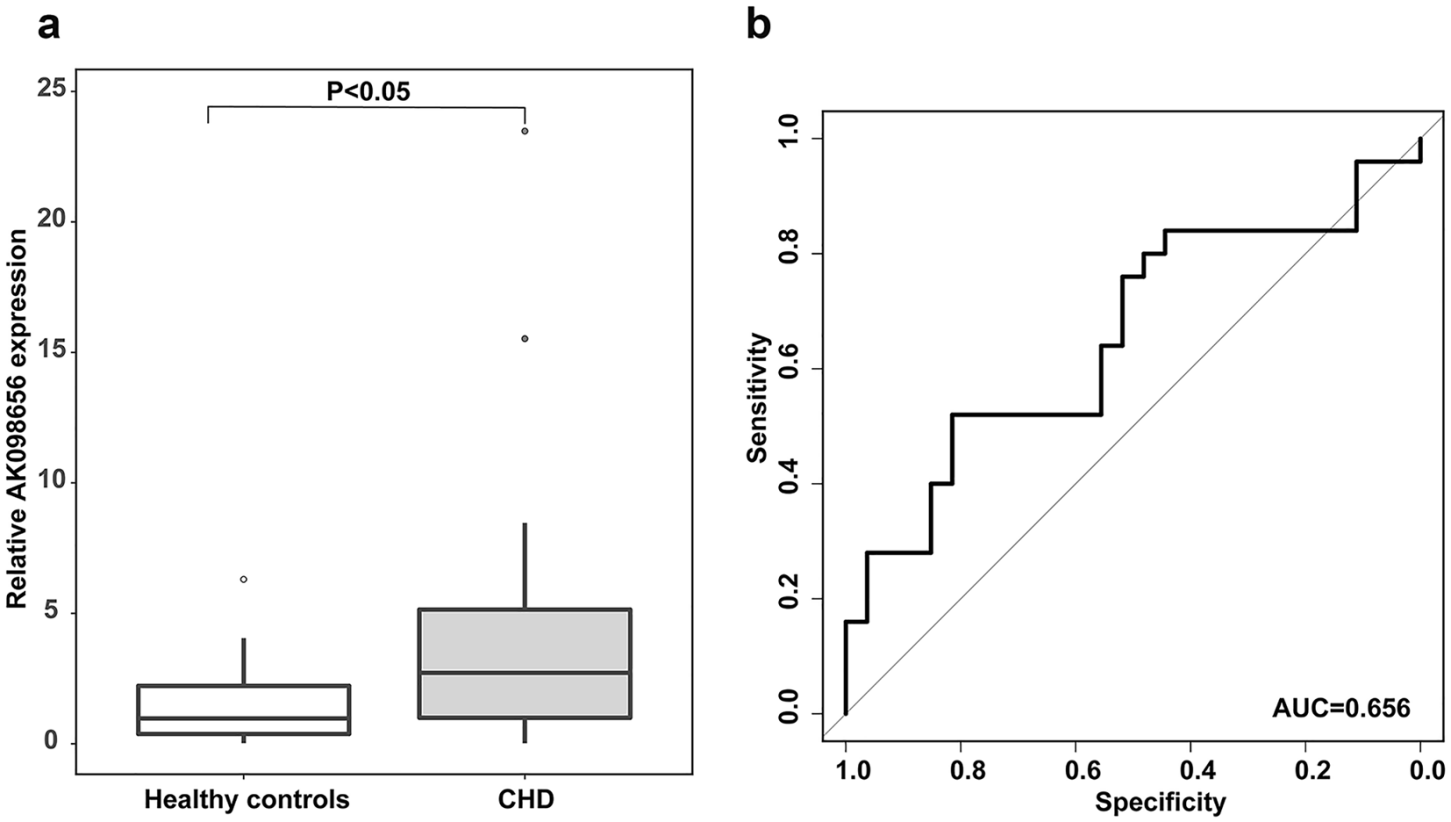
Diagnostic value of AK098656 for CHD. **a** AK098656 expression level in healthy controls and CHD groups. **b** Discrimination ROC for healthy controls and CHD groups

AK098656 expression level was significantly higher in CHD patients compared to healthy controls (P = 0.038; Fig. 1A). In order to quantify the discrimination ability between healthy controls and CHD patients, ROC curve analysis revealed that AK098656 was a valuable predictor for CHD risk (AUC = 0.656, 95% CI 0.501–0.809; Fig. 1B).

### Independent predictive value for AK098656 expression for CHD complication risk

**Fig. 2:**
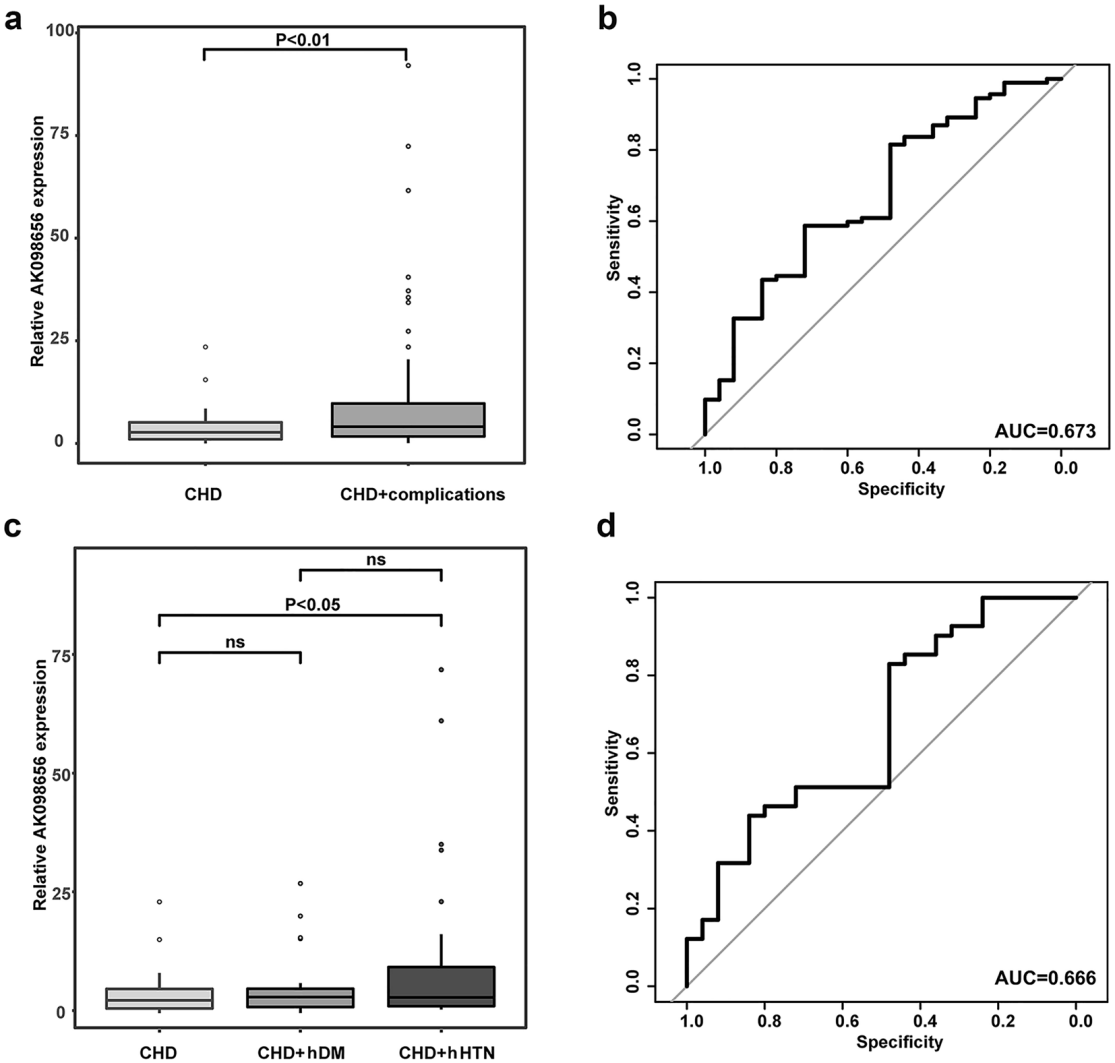
Predictive AK098656 value for CHD patients with complications. **a** AK098656 expression level boxplot for the CHD and CHD + complications groups. **b** Discrimination ROC for the CHD and CHD + complications groups. **c** An AK098656 expression boxplot for the CHD, CHD + hDM, and CHD + hHTN groups. **d** Discrimination ROC for the CHD and CHD + hHTN groups

Because CHD is often accompanied by other chronic complications, such as hypertension and diabetes, AK098656 expression level in CHD and CHD + complications patients was further analyzed. The AK098656 expression level in CHD + complications patients was significantly higher than that in CHD alone patients (P = 0.005; Fig. 2A). The ROC curve analysis revealed that AK098656 had the ability to distinguish between the CHD and CHD + complications groups (AUC = 0.673, 95% CI 0.552–0.793; Fig. 2B). To further analyze the effect of AK098656 on CHD complications, we examined the expression of AK098656 in different groups of CHD complications.   The expression of AK098656 in CHD + hHTN patients was significantly higher than that in CHD alone patients (P = 0.030). In CHD patients with diabetes mellitus, AK098656 expression was slightly higher than that in CHD patients, although no statistical difference was present (P = 0.365). In addition, there was no significant difference in AK098656 expression level between CHD + hDM and CHD + hHTN patients (P = 0.135; Fig. 2C). The ROC analysis implied that AK098656 had the potential to distinguish between CHD and CHD + hHTN groups (AUC = 0.666, 95% CI 0.528–0.805; Fig. 2D).

### AK098656 increases coronary stenosis risk in CHD patients

**Fig. 3:**
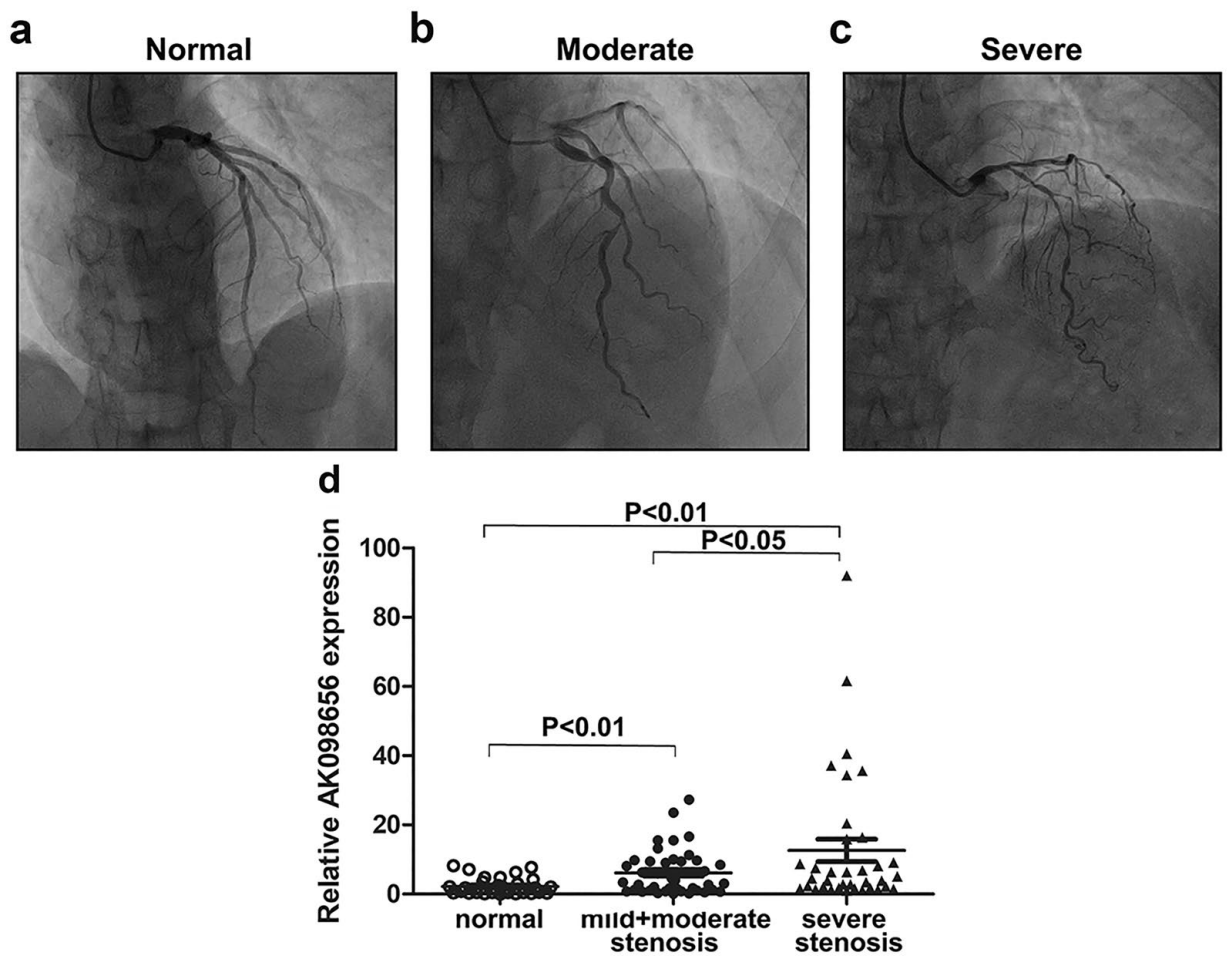
Correlation between AK098656 expression and coronary stenosis. Coronary angiography for **a** normal, **b** mild coronary stenosis, **c** and severe coronary stenosis. **d** The AK098656 expression level in different stenosis degrees (normal, n = 27; mild + moderate, n = 43; and severe, n = 36)

CHD severity was divided into three degrees: mild (stenosis degree for all coronary arteries was < 70%), moderate (stenosis degree of one coronary artery was ≥ 70%), and severe (the stenosis degree of two or more coronary arteries was ≥ 70%; Fig. 3A–C). It is worth noting that AK098656 expression was gradually upregulated with the increase in stenosis degree (Fig. 3D) and was positively associated with stenosis severity (R = 0.261, P = 0.006). It is possible that a higher AK098656 expression may indicate a more severe coronary stenosis.

## Discussion

In this study, human-specific lncRNA AK098656 was significantly increased in CHD patients, especially in those with hypertension. In addition, AK098656 was positively correlated with coronary stenosis severity in CHD patients. This study not only suggested that AK098656 could predict CHD severity, but also proposed that elevated AK098656 in hypertensive individuals may enhance the risk of CHD. The results also confirmed a previous report that AK098656 was upregulated in hypertensive patients compared to normotensive controls [19]. A great deal of research shows that diabetes has a significant effect on coronary arteries and cardiac microcirculation, but also stipulated the idea that elevated. Both insulin resistance and hyperglycemia inflict significant damage to the cardiovascular system [21, 22]. Thus, AK098656 expression was further investigated in CHD + hDM patients. However, AK098656 expression in the CHD + hDM group was not greater than that in the CHD group. An explanation for this observation cannot be suggested based on the current experiment data. However, the insignificant difference between the CHD + hDM and CHD groups may be due to the study’s small sample size.

Differentiation and phenotypic transformation of VSMCs are key initial steps in cardiovascular diseases, such as hypertension, atherosclerosis, and restenosis after angioplasty [23]. Many studies have confirmed that under the influence of different external factors, VSMCs show different phenotypic characteristics. Thus, transformation of different VSMC phenotypes plays an important role in the occurrence and development of a variety of vascular diseases [24]. Therefore, it is initial key steps to investigate the molecular mechanisms of VSMC differentiation and phenotypic transformation for prevention and treatment of these diseases. Differentiated and mature VSMCs mainly maintain their vascular morphology, while dedifferentiated “secretory” VSMCs secrete a large amount of extracellular matrix (ECM) through migration and proliferation. They also play an important role in the occurrence and development of a variety of vascular diseases, including CHD [24, 25]. Angiotensin II and epidermal growth factor are widely recognized to induce proliferation and migration of mature VSMCs [26, 27]. Insulin-like growth factor-1 (IGF-1) is one of the most intensively studied factors that can inhibit phenotypic VSMC transformation. IGF-1 activates the downstream PI3K-AKT signaling pathway by binding to the IGF receptor, thereby increasing the expression of VSMC systolic protein [28]. In addition, tumor growth factor, rapamycin, and microRNA (miR-221, -222, -21, -146A, and -133) are all involved in the regulation of cell proliferation, apoptosis and migration [29, 30]. According to Jin et al. study, transgenic AK098656 rats spontaneously developed hypertensive phenotypes accompanied by reduced lumen diameter and enhanced media/lumen ratio [19]. Then, AK098656 directly bound to MYH11, which was a smooth muscle cell-specific contractile protein. Fibronectin is another AK098656 binding protein that is an essential ECM component [19]. Both MYH11 and fibronectin promote VSMCs plasticity and synthetic phenotype in hypertension pathogenesis [31, 32]. To some extent, the above evidence may explain the mechanism of increased AK098656 expression aggravating coronary artery stenosis, which eventually affects the stenosis score in CHD patients.

Increasing evidence has demonstrated that lncRNAs are not “transcriptional junk”. In fact, lncRNAs play various regulatory roles in each step of gene expression and translation via epigenetic regulation, alternative splicing regulation, or acting as a molecular sponge [33]. A large number of lncRNAs may be part of a complex regulatory network in physiopathology in the cardiovascular system. Consequently, lncRNAs may open up new avenues for diagnosis or serve as therapeutic targets for CHD treatment. For instance, long intergenic noncoding RNA-p21 (lincRNA-p21) has been reported to be down-regulated in patients with CHD, and injection of lentivirus si-lincRNA-p21 at the injured site induced neointimal hyperplasia [16]. LncRNA-MALAT1 mediates endothelial progenitor cells autophagy by activating mTOR signaling pathway and improves coronary atherosclerotic heart disease [34].

There were some limitations in this study. (1) A total of 144 subjects were analyzed in this study, resulting in a relatively small sample size, which may have resulted in insufficient statistical power. (2) Because the clinical data and samples were all collected from a general population at a single center, selection bias may be present due to regional restrictions. Therefore, the results should be further confirmed via large, multi-center, prospective clinical trials. (3) Detailed lncRNA-AK098656 mechanisms in CHD or CHD + complications patients were not investigated.

## Conclusions

In conclusion, our present study found that AK098656 expression in CHD patients was significantly higher than that in healthy controls. The results showed that AK098656 expression in CHD + hHTN and CHD + hDM + hHTN patients was significantly increased compared to CHD alone patients. AK098656 expression was also positively correlated with the degree of coronary artery stenosis. Thus, this study revealed a novel molecule that regulates CHD development.

## Data Availability

All data generated or analyzed during this study are included in this published article.

## Abbreviations

AUC: Area under the curve
CHD: Coronary heart disease
ECM: Extracellular matrix
hDM: Human diabetes mellitus
hHTN: Human hypertension
IGF-1: Insulin-like growth factor-1
lncRNA: Long non-coding (lnc) RNA
VSMCs: Vascular smooth muscle cells
